# Time to antibiotics and outcomes in cancer patients with febrile neutropenia

**DOI:** 10.1186/1472-6963-14-162

**Published:** 2014-04-10

**Authors:** Thomas Perron, Mohamed Emara, Shahid Ahmed

**Affiliations:** 1 Department of Medicine, University of Saskatchewan, Saskatoon, SK, Canada; 2 Saskatoon Cancer Center, Saskatchewan Cancer Agency, University of Saskatchewan, Saskatoon, SK, Canada; 3 Department of Community Health and Epidemiology, University of Saskatchewan, Saskatoon, SK, Canada; 4 Department of Medicine, Site Leader for Gastrointestinal Cancer, Saskatoon Cancer Center, University of Saskatchewan, 20 Campus Drive, Saskatoon, SK S7N4H4, Canada

**Keywords:** Time to antibiotic, Febrile neutropenia, Length of stay, Outcome, Hospital mortality

## Abstract

**Background:**

Febrile neutropenia is an oncologic emergency. The timing of antibiotics administration in patients with febrile neutropenia may result in adverse outcomes. Our study aims to determine time-to- antibiotic administration in patients with febrile neutropenia, and its relationship with length of hospital stay, intensive care unit monitoring, and hospital mortality.

**Methods:**

The study population was comprised of adult cancer patients with febrile neutropenia who were hospitalized, at a tertiary care hospital, between January 2010 and December 2011. Using Multination Association of Supportive Care in Cancer (MASCC) risk score, the study cohort was divided into high and low risk groups. A multivariate regression analysis was performed to assess relationship between time-to- antibiotic administration and various outcome variables.

**Results:**

One hundred and five eligible patients with median age of 60 years (range: 18–89) and M:F of 43:62 were identified. Thirty-seven (35%) patients were in MASCC high risk group. Median time-to- antibiotic administration was 2.5 hrs (range: 0.03-50) and median length of hospital stay was 6 days (range: 1–57). In the multivariate analysis time-to- antibiotic administration (regression coefficient [RC]: 0.31 days [95% CI: 0.13-0.48]), known source of fever (RC: 4.1 days [95% CI: 0.76-7.5]), and MASCC high risk group (RC: 4 days [95% CI: 1.1-7.0]) were significantly correlated with longer hospital stay. Of 105 patients, 5 (4.7%) died & or required ICU monitoring. In multivariate analysis no variables significantly correlated with mortality or ICU monitoring.

**Conclusions:**

Our study revealed that delay in antibiotics administration has been associated with a longer hospital stay.

## Background

Neutropenia is a major dose-limiting toxicity of cytotoxic agents that predisposes cancer patients to serious infections [1-3]. Febrile neutropenia is considered to be an oncologic emergency. The risk of life threatening bacterial infection increases when absolute neutrophil count (ANC) drops below 0.5 × 10^9^/l [1]. Although an infectious source is identified in approximately 20 to 30% of episodes of neutropenia and fever [3], empiric broad spectrum antibiotics are the universal therapy for patients with febrile neutropenia. It involves the initiation of antimicrobial therapy in patients with neutropenia, at the time of the onset of fever, without establishing a definitive diagnosis of microbial infection. A prolonged time-to-antibiotic administration can result in adverse outcomes in immune compromised patients. For instance, the mortality rate, related to a febrile neutropenic episode, has been variably reported between 2% and 20% [2]. Therefore, it is crucial to recognized neutropenic fever early and to commence broad spectrum empiric antibiotics promptly in order to avoid sepsis syndrome and possible death.

The American Society of Clinical Oncology and an international guideline panel of the Surviving Sepsis Campaign recommend administering the first dose of empiric antibacterial therapy as soon as possible after triage (within an hour) to the patients with febrile neutropenia [4,5]. Several studies assessed time-to- antibiotic administration in cancer patients presenting to emergency department (ED) with febrile neutropenia [6-9]. However, limited evidence is available with respect to association between time-to- antibiotic administration and length of hospital stay and mortality in the era of modern anti-cancer therapy. For instance, a national audit of 95 United Kingdom hospitals reported that only 18 to 26% patients with neutropenic fever received initial empiric antibiotics within the one hour “door-to-needle” target time frame with mean mortality rate of 9% [8]. However, relationship between timing of antibiotic administration and mortality or length of hospital stay was not assessed. In another report median time from triage to antibiotic administration, in patients with febrile neutropenia, was 5 hours (range: 1.23–22.8 hours). However, delayed antibiotic administration was not associated with increased risk of death or increased length of hospital stay [9].

The current study aims to determine time-to- antibiotic administration in adult cancer patients with febrile neutropenia, a quality measure in cancer care; and to assess the relationship between time-to- antibiotic administration and length of hospital stay, intensive care unit (ICU) monitoring, and hospital mortality.

### Objectives

•To determine relationship between time-to- antibiotic administration and hospital stay in adult cancer patients with febrile neutropenia treated with chemotherapy.

•To determine relationship between time-to- antibiotic administration and ICU monitoring or hospital mortality in adult cancer patients with febrile neutropenia treated with chemotherapy.

## Methods

The study protocol was approved by the Biomedical Ethics Board of University of Saskatchewan. The study population was comprised of a cohort of consecutive adult cancer patients with a diagnosis of febrile neutropenia who were hospitalized at a tertiary care hospital (the Royal University Hospital) between January 2010 and December 2011. International Classification of Disease (ICD) code was used to identify eligible patients. Diagnosis was verified using Saskatchewan Cancer Registry data. Medical records of all patients were reviewed retrospectively. Information was collected from the hospital and cancer clinic record using a standard abstraction sheet. Eligible patients were meeting the following criteria: diagnosis of malignancy treated by chemotherapy that was causative of or contributive to neutropenia (granulocyte count < 500/μL or is expected to decrease to <500 cells/microL over the next 48 hours), temperature greater than 38°C (measured orally and documented by the patient or the medical/nursing staff), and age greater ≥18 years. Only the first febrile episode occurring in a patient during the study period was considered.

Patients were considered for inclusion if they were admitted from the emergency department, or form an ambulatory care facility (the Saskatoon Cancer Center) to the oncology ward. Time to antibiotic administration was determined by time of ED registration until time of first dose of antibiotic administration as indicated in the medication administration record. For patients who were admitted from the outpatient facility, time of initial assessment by a nurse during clinic visit was used as an indicator of initial awareness of patient being febrile. Patients with fever and neutropenia but no documented malignancy were excluded.

The Multinational Association for Supportive Care in Cancer (MASCC) validated risk index score was used to stratify patients cohort into two risk groups [10,11]. (1) high-risk group (MASCC score <21 points) and (2) low-risk group (score ≥ 21 points). A secondary analysis was performed in patients with solid tumors and with hematological malignancies.

### Statistical analysis

The data are reported as mean, medians, standard deviation and ranges. For descriptive statistics Chi square test and student *t* test were performed. Pearson correlation was done to assess correlation between time-to- antibiotic administration and length of hospital stay. Univariate regression analysis was performed and following clinical variables were examined to assess their association with the duration of hospital stay: time-to- antibiotic administration, age, gender, comorbid illness, advanced disease, high risk disease, hematological malignancy, ANC, blood urea nitrogen (BUN), known source of fever, serum creatinine, abnormal chest x-ray, and prophylactic use of filgrastim and or antibiotic. A multiple linear regression model was developed using variables that significantly correlated with hospital stay (p ≤ 0.05 in univariate analysis), to identify their individual contribution to length of hospital stay. Binary logistic regression analysis was performed, to assess relationship between various clinical variables and a composite outcome of ‘Serious Adverse Events’ defined as hospital mortality and or ICU admission (both variables were not mutually exclusive) . Time to discharge was estimated using Kaplan Meier method. Log Rank test was done for comparison of time to discharge. All two-tailed p-values <0.05 were considered to be significant. The statistical analysis was performed using SPSS version 21 (SPSS Inc. Chicago, IL).

## Results

One hundred and five eligible patients with a median age of 60 years (range: 18–89 years) and M:F of 43:62 were identified. Patients’ characteristics are described in Table 1. Of total 105 patients, 37 (35%) were in MASCC high risk group and 68 (65%) were in MASCC low risk group. Fifty two (49%) patients had a comorbid illness, 46 (44%) had a hematological malignancy, and 63 (60%) had advanced disease. Patients in high risk group were older, predominantly male, and had a significantly higher prevalence of major medical illnesses (Table 1). Patients with a hematological malignancy had more advanced disease and more often received prophylactic filgrastim and or antibiotics, during the last three months, compared with patients with a solid tumor. Of 105 patients, 89 (85%) were presented to the ED and 16 (15%) were hospitalized from the ambulatory clinic. Median time-to- antibiotic administration was 2.5 hrs (0.03-50 hrs). Nine percent patients received antibiotic treatment within an hour of registration and 95% received antibiotics within 9.3 hrs. Eighty-eight (84%) patients were treated with broad spectrum penicillin and 50 (47.6%) patients received prophylactic filgrastim and or antibiotics over the past three months. Median length of stay was 6 days (range: 1–57). A known source of infection was identified in 23 (22%) patients (bacteremia, n = 12, positive urine culture, n = 8, and chest infiltrates, n = 9). Pearson correlation between time-to- antibiotic administration and length of stay was 0.26 (p = 0.008). In univariate analysis time-to- antibiotic administration, MASCC high risk group, known source of fever, and BUN were significantly correlated with length of stay (Table 2). In the multivariate analysis time-to- antibiotic administration (regression coefficient [RC]: 0.31 days [95% CI: 0.13-0.48]), known source of fever (RC: 4.1 days [95% CI: 0.76-7.5]), and MASCC high risk group (RC: 4 days [95% CI: 1.1-7.0]) were significantly correlated with length of stay. Of 105 patients, 5 (4.7%) patients died and or required ICU admission. Four died (3 patients with hematological malignancies and 1 with solid tumors) and 2 required intensive care monitoring. Overall 3 of 17 (17.6%) patients with leukemia died or required ICU admission compared with 2 of 86 (2.3%) patients with non-leukemic malignancy (p = 0.029). No significant difference was noted between the two MASCC risk groups with respect to ICU admission or mortality. In univariate logistic regression analysis, diagnosis of leukemia (odd ratio, 9.2, 95% CI: 1.4-60.1) and bacteremia (odd ratio, 10.3, 95% CI: 1.4-74.1) were significantly correlated hospital mortality and or ICU admission (Table 3). On multivariate analysis no clinical variable significantly correlated with ICU admission or mortality.

**Table 1:** Characteristics of patients treated with febrile neutropenia

**Clinical variables**	**Total patients N = 105 (%)**	**High risk patients N = 37 (%)**	**Low risk patients N = 68 (%)**	**P value**
Median age yrs	60 (18–89)	66 (46–86)	55 (18–89)	0.058
Age over 65	36 (34)	22 (60)	14 (21)	<0.001
Male	43 (41)	21 (57)	22 (32)	0.01
MASCC score	21 ± 2.5	18 ± 1.3	23 ± 1.5	0.15
Comorbid illness	52 (49)	32 (86)	20 (29)	<0.001
Diabetes mellitus	12 (11)	8 (22)	4 (6)	0.02
Chronic renal failure	4 (4)	3 (8)	1 (2)	0.12
Hematological malignancies	46 (44)	19 (51)	27 (40)	0.17
Advanced disease	63 (60)	24 (65)	39 (57)	0.29
Prophylactic filgrastim & or antibiotics	50 (48)	17 (46)	33 (49)	0.48
Mean SBP mmHg	123 ± 22	120 ± 21	124 ± 23	0.71
Mean heart rate bpm	105 ± 20	101 ± 17	107 ± 21	0.97
Mean O_2_ saturation%	97 ± 2	97 ± 2	97 ± 2	0.76
Mean WBC 10^9^/l	0.83 ± 2.8	1.5 ± 4.8	0.99 ± 0.69	0.056
Mean ANC 10^9^/l	0.21 ± 0.23	0.21 ± 0.18	0.21 ± 0.25	0.08
Mean hemoglobin g/L	98 ± 21	97 ± 18	102 ± 23	0.20
Mean platelet count 10^9^/l	136 ± 102	127 ± 106	156 ± 98	0.004
Mean serum sodium mEq/L	135 ± 3	134 ± 3	134 ± 4	0.60
Mean serum creatinine μmol/L	69 ± 36	99 ± 50	68 ± 16	<0.001
Mean BUN mmol/L	5 ± 4.2	8.6 ± 5.3	4.7 ± 2.6	<0.001
Median TAA hrs (range)	2.5 (0.03-50)	3 (0.22-19)	2.5 (0.03-50)	0.16

ANC = absolute neutrphil count, BUN = blood urea nitrogen, TAA = time to antibiotics administration, WBC = white blood cell, ± standard deviation.

**Table 2:** Relationship between duration of hospital stay and clinical variables using univariate regression analysis

**Variables**	**Regression coefficients**	**Standard error**	**P value**
Time to antibiotic	0.26	0.10	0.008
MASCC high risk group	5.1	1.5	0.001
Blood urea nitrogen (BUN)	0.46	0.17	0.009
Known sources of fever	5.3	1.7	0.002
Hematological malignancy	2.1	1.5	0.16
Age	0.10	0.05	0.17
Prophylactic filgrastim and or antibiotics	1.8	1.5	0.21
Comorbid illness	1.8	1.5	0.24
Advanced stage	1.6	1.5	0.29
Absolute neutrophil count	3.2	3.2	0.32
Ambulatory care admission	−1.5	2.0	0.46
Serum creatinine	0.01	0.02	0.66
Female gender	−0.05	1.5	0.97

MASCC = the Multinational Association for Supportive Care in Cancer; A.

**Table 3:** Relationship between important clinical variables and an adverse event in patients with febrile neutropenia

**Variables**	**Risk of mortality/ICU admission (odd ratio)**	**95% confidence interval**	**P value**
Bacteremia	10	1.3-71.5	0.03
Leukemia	9.2	1.4-60.2	0.02
Hematological malignancies	5.5	0.5-51.2	0.13
Age >65 yrs	3.5	0.55-22.6	0.18
Known source	2.5	0.39-15.9	0.33
Male gender	2.5	0.36-14.0	0.38
MASCC high risk	1.2	0.19-7.8	0.82
Time-to-antibiotic	0.79	0.47-1.33	0.38

MASCC = the Multinational Association for Supportive Care in Cancer.

### Duration of hospital stay

**Figure 1:**
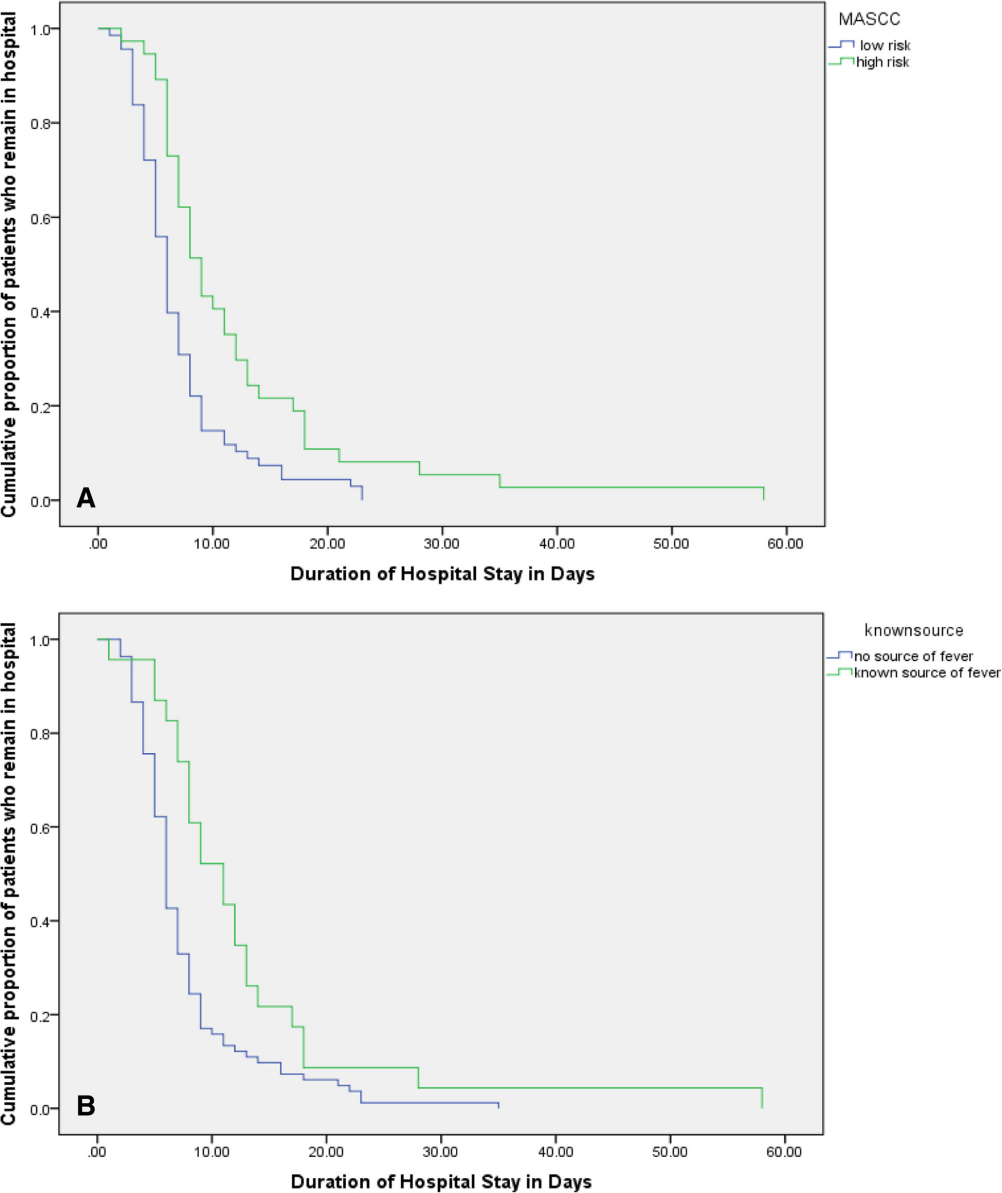
**Duration of hospital stay for both low and high MASCC risk score. (A)** Duration of hospital stay for both known and unknown sources of infection **(B)**.

Median duration of hospital stay in patients with MASCC high risk group was 9 days (95% CI: 7.3-10.7) compared with 6 days (95% CI: 5.3-6.7), in patients with MASCC low risk score p < 0.001 (Figure 1A). Patients with a known source of fever and documented infection had median duration stay of 11 days (95% CI: 7.5-14.5) compared with 6 days (95% CI: 5.5-6.5) in patients with no documented source of fever, p = 0.004 (Figure 1B).

With respect to underlying malignancy, median duration of hospital stay in patients with hematological malignancies was 8 days (95% CI: 6.7-9.2) compared with 6 days (95% CI: 5.1-6.8) in patients with solid tumors, p = 0.13. No significant difference in the length of hospital stay was noted in patients with underlying leukemia versus patients with non-leukemic hematological malignancies.

## Discussion

Our study demonstrated a positive relationship between a delay in antibiotic administration and a longer hospital stay. An hour delay in antibiotic administration was resulted in approximately eight-hours increase in length of hospital stay. The study also revealed a broad range of times until antibiotic administration. Several guidelines advocate initiation of empiric antimicrobial therapy within 60 minutes of presentation in patients with febrile neutropenia [4,5]. Although 95% patients received first dose of empiric antibiotic therapy within 10 hours of registration, only 9% received treatment within an hour. Delay over 5 hrs was noted in 20% patients, with longest being 50 hrs. The causal relationship between delay in time-to-antibiotics and prolong hospital stay in our study cohort remains speculative and may have been due to slow recovery from underlying infection, persistent fever and other complications. The common reasons for delayed in assessment and antibiotic administration reported in literature are: Antibiotics are prescribed by doctors but their administration is delayed, prolonged time to assessment by the junior staff, lack of awareness of natural evolution of course of neutropenic sepsis, failure of the emergency department to stock the appropriate antimicrobial therapy, and lack of a distinct protocol for febrile neutropenia [8,9]. A significant decrease in time-to- antibiotic administration has been demonstrated when order sets were utilized to increase efficiency in antibiotic administration or when multiple ED initiatives were taken to improve patients triage [12-14].

In addition to time-to- antibiotic administration, MASCC high risk score and known sources of fever were correlated with delay in hospital discharge. Patients in high risk MASCC group had 4 days longer hospital stay compared with low risk patients with MASCC score of ≥21. The MASCC risk index score was developed with the aim to identify patients with chemotherapy-induced febrile neutropenia who are at low risk of serious medical complication development [10,15]. In a multinational, multicenter study of more than 1,100 patients with febrile neutropenia, the Multinational Association for Supportive Care in Cancer (MASCC) demonstrated that certain characteristics such as old age, disease burden, hypotension, dehydration, obstructive lung disease, hematological malignancy, and in-patients status are associated with high risk of serious medical complications, and absence of such features can identify low-risk patients. MASCC risk index has been validated and has become part of the selection process of patients who can safely be treated at home [15].

Of note patients who had a known source of fever had 4 days longer length of stay compared with patients with no known source of fever. An infectious source is indentified only in about one third of patients with FN [1]. Bacteria are the most frequent infectious causes of neutropenic fever and bacteremia is often the only evidence of infection. More than two third of identified infections are thought to arise from the patient’s endogenous flora. Immunosuppressive effects of anti-cancer therapy, underlying cancer and its associated humoral or cellular immune deficits predispose for specific types of infections [1-3]. For example, T cell defects associated with lymphoproliferative disorders result in an increased risk of infection with intracellular pathogens, such as Listeria monocytogenes. Although antibiotics are empirically administered, it should always include appropriate coverage for suspected organisms. The empiric antibiotic therapy later can be modified once updated clinical and microbiologic information are available.

Although one mmol/l rise in BUN resulted in approximately 11 hours longer length of stay, after adjustment for MASCC score that takes dehydration into account, elevated BUN was not independently correlated with prolong hospital stay. Elevated BUN is a marker of hypovolemia and has been identified as independent prognostic factors for mortality and hospital stay in critically ill patients [16]. Patients with cancer who are undergoing active treatment can have reduced oral intake and are at risk of dehydration due to anorexia, early satiety, nausea and vomiting, mucositis, dysphagia, delayed gastric emptying, and diarrhea. Hence, adequate hydration is crucial in patients with febrile neutropenia to avoid renal function impairment, longer stay and other complications.

The mortality rate of the study cohort was 3.8%. Patients with prolonged neutropenia, major comorbid illness and organ dysfunction are at risk of serious complications [1,5,17]. A very high mortality rate, up to 90%, had been reported in patients with febrile neutropenia before the empiric use of antibiotic [18]. Kuderer and others, using longitudinal discharge database derived from 115 US hospitals, studied more than 4,000 adult cancer patients with febrile neutropenia who were hospitalized between 1995–2000, and reported in-hospital mortality of 9.5% [2]. Underlying cancer, major comorbid illness, and infectious complications such as pneumonia and sepsis were significantly associated with increased mortality. Patients who are critically ill have poor outcomes and high hospital mortality. For example, a French study involving neutropenic patients with severe sepsis or septic shock, treated between 1998 and 2008, demonstrated hospital mortality rate of 50% [19]. In our study patients with underlying leukemia or bacteremia were at high risk of hospital mortality and or ICU admission, however, no factor independently correlated with an adverse outcome after adjustment for other variables. Likewise, time-to- antibiotic administration did not correlate with ICU admission or mortality. It is plausible that due to small number of serious adverse events, our study was not adequately powered to detect significant association between ICU admission or hospital mortality and various prognostic variables.

The current study is one of the very few studies that evaluated relationship between timing of antibiotic and duration of hospital stay and other important outcomes using a validated risk score. One of the limitations of the present study is that it did not evaluate potential reasons for delayed in assessment and antibiotic administration. Furthermore, patients who were treated at the rural hospitals were excluded; hence, the results may not be generalizable to the rural cancer population. Although most patients developed fever at home, information on the time when fever was first noted by the patients was not available in most cases and registration time was used as surrogate of onset of fever to eliminate information bias.

## Conclusions

This retrospective cohort study revealed a positive relationship between time-to-antibiotic and prolonged hospital stay. Only one in ten patients received antibiotic within an hour of registration. In addition, high risk patients or patients with known source of infection had longer hospital stay. The study with its limitation did not reveal association between delay in antibiotic administration and hospital mortality or ICU admission. We believe that patients and health care staff education, establishment of bench mark time, and access to standard treatment protocol in ED can potentially be helpful for prompt triage of patients with febrile neutropenia and timely antibiotic administration to shorten hospital stay and avoid other complications.

## Abbreviations

ANC: Absolute neutrophil count; ED: Emergency department; FN: Febrile neutropenia; ICU: Intensive care unit.

## Competing interests

The authors have no financial or non-financial interests in relation to the manuscript to disclose.

## Authors’ contributions

SA has made contribution to 1) conception and design, 2) analysis and interpretation of data; and 3) drafting the manuscript and final approval of the version to be published. ME made contribution to acquisition of additional data, drafting and final approval of manuscript. TP has made substantial contributions to acquisition of data, and has been involved in drafting the manuscript final approval of the version to be published.

## Pre-publication history

The pre-publication history for this paper can be accessed here:

http://www.biomedcentral.com/1472-6963/14/162/prepub
